# Draft Genome Sequence of Pandoravirus japonicus Isolated from the Sabaishi River, Niigata, Japan

**DOI:** 10.1128/MRA.00365-21

**Published:** 2021-05-13

**Authors:** Nao Hosokawa, Haruna Takahashi, Keita Aoki, Masaharu Takemura

**Affiliations:** a Laboratory of Biology, Institute of Arts and Sciences, Tokyo University of Science, Shinjuku, Tokyo, Japan; b Laboratory of Biology, Graduate School of Mathematics and Science Education, Tokyo University of Science, Shinjuku, Tokyo, Japan; DOE Joint Genome Institute

## Abstract

“*Pandoraviridae*” is a proposed family of the phylum *Nucleocytoviricota*. Its features include an amphora-shaped capsid and the largest genome among all viruses. We report the isolation and genome sequencing of a new member of this family, named Pandoravirus japonicus, the third strain discovered in Japan.

## ANNOUNCEMENT

Many members of the proposed family “*Pandoraviridae*” have previously been reported (1–7). Here, we report the isolation of a new member, Pandoravirus japonicus, from a freshwater sample (taken in a 50-ml tube) from the mouth of the Sabaishi River in Niigata, Japan (37°23′21.7″N, 138°33′59.4″E).

**FIG 1:**
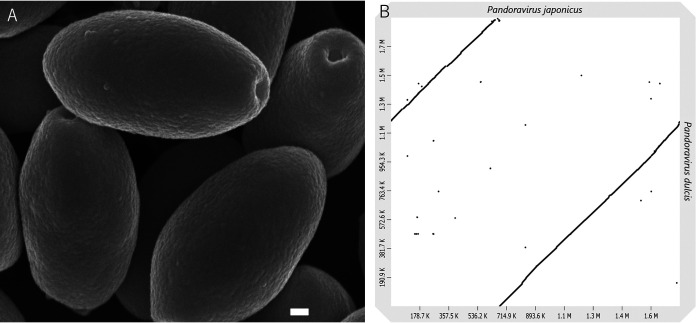
(A) Scanning electron microscopy (SEM) image of *Pandoravirus japonicus*. Acanthamoeba castellanii cells were cultured in PYG medium in 75-cm^2^ culture flasks and infected with *P. japonicus*. Four days after infection, viral particles in the medium were collected at 8,000 × *g* for 35 min at 4°C. The virus pellet was resuspended in 5 ml of phosphate-buffered saline (PBS). After centrifugation (8,000 × *g* for 35 min at 4°C), the virus pellet was resuspended in 500 μl of 2% glutaraldehyde (GA) in PBS, followed by centrifugation, resuspension, and fixation with 50 μl of 2% GA in PBS, and postfixation in 2% osmium tetra-oxide for 2 h in the ice bath. Then, the specimens were dehydrated with graded ethanol and CO_2_ critical point drying. The dried specimens were coated by an osmium plasma ion coater. SEM was performed using a model JSM-7500F microscope (JEOL Ltd., Tokyo, Japan), and secondary electron images at 5 kV were captured at the Hanaichi UltraStructure Research Institute (Aichi, Japan). Bar, 100 nm. (B) Dot plot analysis for genome comparison of *P. japonicus* and *P. dulcis* using D-GENIES with default parameters (9).

The sample (4.5 ml) was mixed with 4.5 ml of 2× peptone-yeast-glucose (PYG) medium, 50 μl of Acanthamoeba castellanii (amoeba) cells (1.5 × 10^5^ cells), and 360 μl of antibiotic solution (8). This mixture was added to a 96-well plate (100 μl per well). After 3 days of culture at 26°C, 10 μl supernatant from each well showing microscopic evidence of cytopathic effects (CPE; delayed proliferation or cell rounding) was serially diluted to 10^11^-fold with PYG medium. Then, 10 μl of each dilution was mixed with 90 μl of PYG medium (16 ml PYG medium and 5.0 × 10^3^ amoeba cells) in a 96-well plate. After several days, amoeba cells (1.5 × 10^6^) were inoculated with the supernatants from wells showing CPE with the highest dilution in a 25-cm^2^ culture flask. One of the supernatants (25 ml) of the amoeba cell culture showing CPE was harvested. Viral particles were collected by centrifugation at 8,000 × *g* for 35 min at 4°C. The resulting pellets were resuspended and washed with 1 ml phosphate-buffered saline (PBS). The preparation was examined using scanning electron microscopy (Fig. 1A). Viral DNA was extracted using the NucleoSpin tissue XS kit (Macherey-Nagel GmbH & Co. KG, Duren, Germany) according to the manufacturer’s instructions. A 10-μl library was prepared using a PacBio (Menlo Park, CA, USA) DNA template prep kit 1.0 (for 3 to 20 kb). SMRTbell templates were annealed using the PacBio DNA/polymerase binding kit P6. The PacBio DNA sequencing kit 4.0 and 8 single-molecule real-time (SMRT) cells were used for sequencing. FALCON software was used to assemble 1,043,248,645 raw reads into one contig with a length of 1,787,274 nucleotides. The reads were mapped to the genomic sequence using CLC Genomics Workbench software ver. 20.0.4 (Qiagen N.V., Hilden, Germany), resulting in 63 single-nucleotide insertions or deletions. The average coverage was 316.71×. These regions were confirmed via capillary sequencing, which allowed us to assemble a final genomic sequence of 1,787,268 bp. The GC content of the genome was 64.0%. Dot plot analysis (9) suggested that a part of the *P. japonicus* genome translocated compared with other pandoravirus genomes, although the possibility of genome rotation due to random origin or misassembly could not be excluded (Fig. 1B). Gene prediction was performed using the GeneMarkS (10) and FgenesV tools. tRNA genes were identified using the tRNAscan-SE server ver. 2.0 (11). All tools described above were run with default parameters unless otherwise specified. We identified 1,361 open reading frames (ORFs) and one tRNA (Ser). Homology searches were performed using BLASTp against the NCBI nonredundant (nr) GenBank database with an E value threshold of <10^−5^, and the gene annotation was manually revised. Most ORFs were highly homologous to other pandoraviruses, and 410 were putative ORFans.

### Data availability.

The sequence data are available in the DDBJ (accession number DRA011890) and GenBank (accession number LC625835).

## References

[1] Philippe N, Legendre M, Doutre G, Couté Y, Poirot O, Lescot M, Arslan D, Seltzer V, Bertaux L, Bruley C, Garin J, Claverie J-M, Abergel C. 2013. Pandoraviruses: amoeba viruses with genomes up to 2.5 Mb reaching that of parasitic eukaryotes. Science 341:281–286. doi:10.1126/science.1239181. 23869018

[2] Scheid P. 2016. A strange endocytobiont revealed as largest virus. Curr Opin Microbiol 31:58–62. doi:10.1016/j.mib.2016.02.005. 27016694

[3] Dornas FP, Khalil JYB, Pagnier I, Raoult D, Abrahao J, La Scola B. 2015. Isolation of new Brazilian giant viruses from environmental samples using a panel of protozoa. Front Microbiol 6:1086. doi:10.3389/fmicb.2015.01086. 26500630 PMC4594340

[4] Andrade ACDSP, Arantes TS, Rodrigues RAL, Machado TB, Dornas FP, Landell MF, Furst C, Borges LGA, Dutra LAL, Almeida G, Trindade GDS, Bergier I, Abrahão W, Borges IA, Cortines JR, de Oliveira DB, Kroon EG, Abrahão JS. 2018. Ubiquitous giants: a plethora of giant viruses found in Brazil and Antarctica. Virol J 15:22. doi:10.1186/s12985-018-0930-x. 29368617 PMC5784613

[5] Aherfi S, Andreani J, Baptiste E, Oumessoum A, Dornas FP, Andrade ACDSP, Chabriere E, Abrahao J, Levasseur A, Raoult D, La Scola B, Colson P. 2018. A large open pangenome and a small core genome for giant pandoraviruses. Front Microbiol 9:1486. doi:10.3389/fmicb.2018.01486. 30042742 PMC6048876

[6] Legendre M, Fabre E, Poirot O, Jeudy S, Lartigue A, Alempic J-M, Beucher L, Philippe N, Bertaux L, Christo-Foroux E, Labadie K, Couté Y, Abergel C, Claverie J-M. 2018. Diversity and evolution of the emerging Pandoraviridae family. Nat Commun 9:2285. doi:10.1038/s41467-018-04698-4. 29891839 PMC5995976

[7] Akashi M, Takemura M. 2019. Co-isolation and characterization of two pandoraviruses and a mimivirus from a riverbank in Japan. Viruses 11:1123. doi:10.3390/v11121123. PMC6950457 31817274

[8] Aoki K, Hagiwara R, Akashi M, Sasaki K, Murata K, Ogata H, Takemura M. 2019. Fifteen marseilleviruses newly isolated from three water samples in Japan reveal local diversity of Marseilleviridae. Front Microbiol 10:1152. doi:10.3389/fmicb.2019.01152. 31178850 PMC6543897

[9] Cabanettes F, Klopp C. 2018. D-GENIES: dot plot large genomes in an interactive, efficient and simple way. PeerJ 6:e4958. doi:10.7717/peerj.4958. 29888139 PMC5991294

[10] Besemer J, Lomsadze A, Borodovsky M. 2001. GeneMarkS: a self-training method for prediction of gene starts in microbial genomes. Implications for finding sequence motifs in regulatory regions. Nucleic Acids Res 29:2607–2618. doi:10.1093/nar/29.12.2607. 11410670 PMC55746

[11] Lowe TM, Chan PP. 2016. tRNAscan-SE On-line: integrating search and context for analysis of transfer RNA genes. Nucleic Acids Res 44:W54–W57. doi:10.1093/nar/gkw413. 27174935 PMC4987944

